# Face masks disrupt holistic processing and face perception in school-age children

**DOI:** 10.1186/s41235-022-00360-2

**Published:** 2022-02-07

**Authors:** Andreja Stajduhar, Tzvi Ganel, Galia Avidan, R. Shayna Rosenbaum, Erez Freud

**Affiliations:** 1 Department of Psychology and the Centre for Vision Research, York University, Toronto, Canada; 2 Department of Cognitive and Brain Sciences, Ben-Gurion University of the Negev, 8410501 Beer-Sheva, Israel; 3 Department of Psychology, Ben-Gurion University of the Negev, 8410501 Beer-Sheva, Israel; 4 Rotman Research Institute, Baycrest Health Sciences, Toronto, Canada

**Keywords:** Face perception, Holistic processing, COVID-19, Inversion effect, Masks

## Abstract

Face perception is considered a remarkable visual ability in humans that is subject to a prolonged developmental trajectory. In response to the COVID-19 pandemic, mask-wearing has become mandatory for adults and children alike. Recent research shows that mask-wearing hinders face recognition abilities in adults, but it is unknown if the same holds true in school-age children in whom face perception is not fully developed. Here we tested children (*n* = 72, ages 6–14 years old) on the Cambridge Face Memory Test – Kids (CFMT-K), a validated measure of face perception performance. Faces were presented with or without masks and across two orientations (upright/inverted). The inclusion of face masks led to a profound deficit in face perception abilities. This decrement was more pronounced in children compared to adults, but only when task difficulty was adjusted across the two age groups. Additionally, children exhibited reliable correlations between age and the CFMT-K score for upright faces for both the mask and no-mask conditions. Finally, as previously observed in adults, children also showed qualitative differences in the processing of masked versus non-masked faces. Specifically, holistic processing, a hallmark of face perception, was disrupted for masked faces as suggested by a reduced face-inversion effect. Together, these findings provide evidence for substantial quantitative and qualitative alterations in the processing of masked faces in school-age children.

**Supplementary Information:**

The online version contains supplementary material available at 10.1186/s41235-022-00360-2.

## Significance statement

Mask-wearing is an effective tool in reducing the novel coronavirus transmission and became prevalent in diverse social contexts including culture events, public transportation, and educational institutions. Previous research showed that masks hinder face perception ability and also change the way faces are processed: relative to unmask faces, the holistic processing of masked faces is severely reduced. Notably, school-age children constantly interact with masked peers and teachers, but it is not clear whether masks hinder their face perception abilities to a similar extent. Here, we address this gap by testing school-age children using the children-adjusted version of a canonical face recognition measure (The Cambridge Face Memory Test-K). We provide empirical evidence that compared with adults, children’s face perception is more negatively impacted by the inclusion of masks. We also find evidence for a reduced holistic processing of the masked faces across ages. In conclusion, our study finds qualitative and quantitative changes in the processing of masked faces among school-age children and adults.

## Introduction

Faces are among the most significant visual stimuli in human perception. A quick glance at a person’s face reveals a plethora of socially relevant information, including their race, age, gender, and emotional state (Tsao & Livingstone, 2008). In response to the COVID-19 pandemic, governments around the world have mandated mask-wearing in public spaces in an effort to curb virus transmission (Canada, 2020). Mask-wearing became mandatory for children and adults alike and was presented as a necessary step to enable the safe re-opening of educational institutions. Recent research has demonstrated that masks hinder face processing abilities in adults, including the ability to perceive the identity of faces (Carragher & Hancock, 2020; Freud et al., 2020) their emotional expression (Calbi et al., 2021), and to recognize voices (Mheidly et al., 2020). The occlusion of the lower part of the face is also expected to hinder face processing abilities in children (for example, see Carbon & Serrano, 2021 that recently showed that children are impaired in their ability to recognize emotions from masked faces), however the extent of this impairment is yet to be determined.

Typical human face perception is characterized by a holistic processing, which emphasizes processing the face as an entire unit rather than relying on its specific features (Farah et al., 1998). Previous research has shown a relationship between face perception abilities and the degree of holistic processing in adults. In particular, face recognition accuracy was found to be correlated with different measures of holistic processing of faces (Richler et al., 2011; Wang et al., 2012; but see Konar et al., 2010 for different findings). The importance of holistic processing for face perception is further emphasized by neuropsychological evidence from both acquired and congenital prosopagnosia, where impairments in face perception abilities are accompanied by alterations of holistic processing (Avidan et al., 2011; Ramon et al., 2010; Tanzer et al., 2013). Indeed, even in typical observers, experimental manipulations that disrupt holistic processing, such as face inversion (Face Inversion Effect, FEI; Yin, 1969; but see Richler et al., 2011) and face alignment (Composite Effect; Young et al., 2013), lead to a robust decrement in face perception abilities.

Face masks conceal the lower half of the face (e.g., the mouth and part of the nose area), making it difficult to process the face in a holistic manner. In accordance with the terminology suggested by Maurer et al. (2002), masks can interfere with the detection of first-order relations that define faces (for example, two eyes above a nose and mouth), with the integration of those features into a coherent gestalt and, more importantly, with the processing of the second-order, fine-grained spatial relations between the features. Consistent with this logic, a number of studies showed reduction in face recognition performance due to disruptions in holistic processing with partially occluded faces (Carragher & Hancock, 2020; Kret & De Gelder, 2012; but see Ruba & Pollak, 2020). Recent studies conducted during the COVID-19 pandemic similarly found that face masks interfere with holistic processing and lead to a reduced face inversion effect (Freud et al. 2020, 2021).

Partial occlusion, as occurs with face masks, and even selective blurring of certain facial features have long been shown to disrupt holistic face processing. Studies have shown that judgements of sex and familiarly by adult participants are hindered when facial features like the nose are masked or manipulated (i.e., outstretched), as the obstruction and manipulation of critical facial features hinders encoding of topographical and textural information about the face and featural interrelationships (Bruce et al., 1993; Roberts & Bruce, 1988). The importance of salient internal facial features like the mouth, nose, and particularly the eyes to the configural processing and successful encoding of faces is further supported in studies that have manipulated interocular distance (Leder et al., 2001) and masked these critical regions (Ellis et al., 1979; Goldstein & Mackenberg, 1966; McKelvie, 1976; Young, 1984; Young et al., 1985).

Despite the wealth of research on the correspondence between holistic processing and face perception in adults, the developmental trajectory of this correspondence has not been directly addressed. Previous studies reported that children’s face perception abilities generally develop slowly, improving precipitously between the ages of 4–11 (Bruce et al., 2000; Geldart et al., 2002) but only showing adult-like levels in performance in adolescence, after years of experience differentiating faces (Carey et al., 1980; Mondloch et al., 2002). Other studies, however, show evidence of adult-like holistic face processing in children as young as four years of age (Cassia et al., 2009; de Heering et al., 2007; Meinhardt-Injac et al., 2017; Pellicano & Rhodes, 2003). Nevertheless, the emerging view is that face perception mechanisms are already present at birth (at least partially) and mature throughout childhood, along the development of cognitive factors that support face perception, such as memory and attention (McKone et al., 2012; see Weigelt et al., 2014).

Given a gradual refinement in face perception abilities from early childhood to adolescence, we predicted that children will be adversely affected by face masks similar to, or even more than adults. We also predicted that face masks will alter holistic processing in children as was previously observed for adults. To test these predictions, we used the Cambridge Face Memory Test-Kids (CFMT-K; Dalrymple et al., 2012), which is considered a reliable test of face recognition abilities in children. The main advantage of using this test for children is that its difficulty has been adjusted from the adult version of the CFMT test, making it a perfect candidate for comparing the effects of face masks across the two populations. In this test, children are asked to recognize children’s faces across increasing levels of difficulty. We generated an adjusted version of the test which included face masks and compared performance in children who completed the masked version of the test with those who completed the unmasked (standard) version. To examine whether any reduction in face perception is accompanied by a qualitative change in holistic face processing, we constructed upright and inverted versions of the CFMT-K and administered them to both groups of children.

## Methods

### Participants

Table 1 summarizes the demographic details of the participants across the different conditions. Seventy-two participants (33 females) with a mean age of 10.7 (SD = 2.3, range 6–14) were recruited using snowball sampling during the period of November/December 2020. This age range was chosen as it covers the age range of elementary school children in Canada. Participants were randomly assigned to the mask/no-mask condition and were compensated for their time ($10 CAD Amazon gift card for 15 min). Thirty-seven participants (19 females) with a mean age of 10.6 (SD = 2.5, range 8–10) were randomly assigned to the masked condition and thirty-five participants (14 females) with a mean age of 10.7 (SD = 2.1, range 7–10) were randomly assigned to the non-masked condition. All participants and their parents/legal guardians provided informed consent prior to participating in the experiment.

**Table 1:** Demographic details of participants for the different experimental conditions

	Children CFMT-K		Adults CFMT		Adult CFMT-K	
	Masked	Non-masked	Masked	Non-masked	Masked	Non-masked
*N* (female)	37 (19)	35 (14)	248 (128)	247 (124)	36 (19)	36 (18)
Age (SD)	10.7 (2.5)	10.7 (2.1)	25.4 (7.6)	27.1 (9.7)	28.2 (5.7)	28.86 (7.0)

A group of 495 adult participants with a mean age of 26.3 years (SD = 8.7, range 18–66) was recruited online (https://www.prolific.co/) during the period of January 2021 and completed the standard CFMT (see details below). Participants were randomly assigned to the mask/no-mask condition and were compensated for their time (~ $6 CAD for 25 min).

Finally, an additional group of 72 adults (37 females) with a mean age of 28.5 years (SD = 6.4, range 18–44) was recruited online (https://www.prolific.co/) during the month of April 2021 and completed the CFMT-K. Participation in the experiment was restricted to participants living in Canada and only those who fall between the ages of 18–45. An equal number of participants participated in both the masked and non-masked conditions (masked condition: *M*_age_ = 28.2, SD = 5.7, range 19–42; non-masked condition: *M*_age_ = 28.8, SD = 7.0, range 18–44), and none of the participants partaking in this experiment were previously tested in January 2021.

All experiments were performed in accordance with relevant guidelines and regulations according to the protocol approved by the ethics review board. All participants provided informed consent. Data and analysis code are available on the Open-Source Framework (https://osf.io/yj38h/) under CC-By Attribution 4.0 International license.

### Materials

The CFMT-K (Dalrymple et al., 2012) was used to assess face perception abilities in the group of children and in one group of adults. The CFMT-K is based on the adult version of the task (Duchaine & Nakayama, 2006). Unlike the adult version, the CFMT-K is shorter and uses children’s faces instead of adult faces. The CFMT-K includes three phases (total of 48 trials) with increasing levels of difficulty. Prior to the beginning of the task, participants are presented with a practice trial with one target cartoon face shown from three different viewpoints, followed by a three-alternative forced-choice task (3-AFC). The first phase (easy) involves learning to recognize four unfamiliar male faces from three different viewpoints (right, front, left) and subsequently testing recognition of these faces in a three-AFC. The second phase (medium) involves a refresher of the four targets presented together from one viewpoint (frontal) followed by testing from novel viewpoints and different lighting conditions. The third phase (difficult) is similar to the second phase but includes test images with added visual noise. The adult version of the CFMT is identical in structure to the CFMT-K, except for the use of adult faces instead of children’s faces and an additional two targets (total of six target faces; total 72 trials).

Participants were randomly assigned to one of two groups. The first group completed the original CFMT (faces without masks), while the second group completed a modified version of the CFMT in which an identical face mask was added to all faces. To explore holistic processing of faces with and without masks, each participant completed the test twice, once with upright faces and once with inverted faces. Block order (upright/inverted) was counterbalanced between participants.

### Procedure

**Fig. 1:**
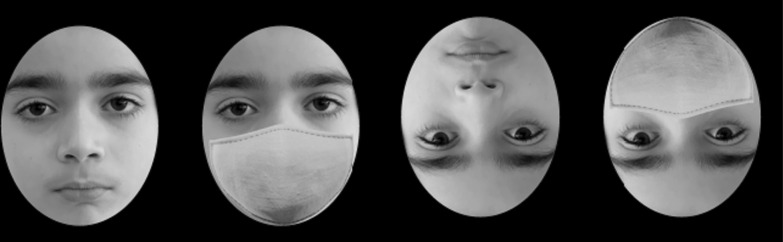
Examples of masked and unmasked faces similar to those used in the experiment. Faces were presented in upright and inverted orientations to evaluate differences in holistic processing associated with inversion and mask wearing. The picture was taken and published with permission from the child and their legal guardians

The CFMT-K was built using jsPsych, an open-source JavaScript plugin library (de Leeuw, 2015), and was hosted on Pavlovia (https://pavlovia.org/). The parents of the children were contacted first via email to obtain consent for their child’s participation. Participants completed the experiment at home and were emailed an experiment link which they could access at any time to complete the experiment. Participants were instructed to complete the experiment independently; for children under the age of 10, parents/legal guardians were encouraged to help their children read the experiment instructions. Participants were randomly assigned to one of two groups. The first group completed the CFMT-K with non-masked faces, while the second group completed a modified version of the CFMT-K in which an identical face mask was added to all faces (Fig. 1). To explore whether holistic processing was employed on faces with and without face masks, each participant completed the task twice, once with upright faces and once with inverted faces. Block order (upright/inverted) was counterbalanced between participants. Accuracy scores (0%–100%) for the upright and inverted faces were computed and served as the dependent variable. Data was processed using Python and statistical analyses were conducted using JASP (*JASP Team*, 2020).

## Results

We explored the extent to which face masks impaired face recognition abilities. To this end, participants completed the CFMT-K with upright and inverted faces (within-subject) while the faces were either masked or non-masked (between-subjects). Participant sex/gender also served as a between-subject variable, as previous research has documented an advantage in face recognition abilities in female participants (Herlitz & Lovén, 2013). In the first two sections below, we report the results from the children group. In the third section, we compare the children to two groups of adults to estimate whether the mask effect was modulated in older ages.

**Fig. 2:**
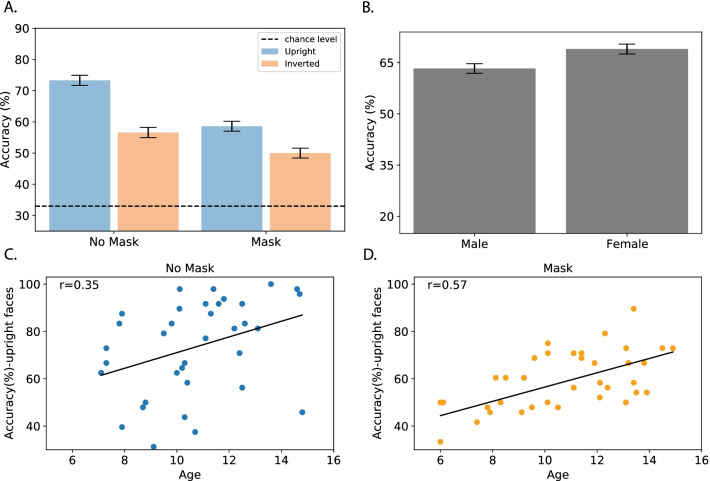
**a** Results of the CFMT-K experiment for non-masked and masked faces across orientations. The dashed horizontal line represents chance level (33%). Performance was significantly impaired for masked faces. An inversion effect was found for masked and non-masked faces, but it was significantly reduced for masked faces. Error bars represent the 95% confidence interval for the main effect of group (mask/no mask). **b** Average performance of males and females on the CFMT-K. Females showed better face recognition abilities than males. Error bars represent the 95% confidence interval for the main effect of gender. (c) Correlation between age and CFMT-K % accuracy for upright non-masked and (d) masked faces. A positive correlation between age and face recognition abilities was found for both conditions, such that face recognition abilities improve with age

Figure 2a shows the group averages across conditions on the CFMT-K. We found a robust alteration in face recognition abilities for masked compared to non-masked faces, such that for upright masked faces there was a decrease of about 20% in the CFMT-K score. Consistent with previous studies, a strong inversion effect was observed for the no-mask condition. This effect was also observed for the masked condition, albeit to a lesser degree.

A repeated measures ANOVA with mask type (mask/no-mask) and orientation (upright/inverted) showed a main effect of mask [*F*_(1,68)_ = 14.31, *p* < 0.001, *η*_*p*_^2^ = 0.17]. The mask effect was accompanied by a strong inversion effect [*F*_(1,68)_ = 55.31, *p* < 0.001, *η*_*p*_^2^ = 0.44] reflecting the well-documented advantage for upright faces.

Importantly, these main effects were qualified by a two-way interaction between face orientation and group [*F*_(1,68)_ = 5.38, *p* = 0.02, *η*_*p*_^2^ = 0.07]. Planned comparison showed that the face inversion effect (FIE) was evident for both non-masked [mean FIE: 23%; *F*_(1,68)_ = 31.74, *p* < 0.001] and masked faces [mean FIE: 15%; *F*_(1,68)_ = 23.16, *p* < 0.001], but it was significantly smaller for the latter, pointing to a qualitative difference in the processing of masked faces. In particular, the size of the inversion effect is suggested to reflect the extent of holistic processing of faces, hence a reduced inversion effect reflects a shift toward a more local/analytical processing (Farah et al., 1995). Importantly, the reduced inversion effect for masked faces could not be attributed to a floor effect, as performance for inverted masked faces was well above chance level (average score for inverted mask faces = 50%, SD = 12; One-sample t-test against chance level (33%)—*t*_(36)_ = 4.86, *p* < 0.001, *η*_*p*_^2^ = 0.79).

An additional main effect of sex/gender was found, with females outperforming males [*F*_(1,68)_ = 7.44, *p* < 0.01, *η*_*p*_^2^ = 0.09; Fig. 2b]. This result is consistent with some of the previous literature (e.g., Rehnman & Herlitz, 2006; but see Grüsser et al. (1985) for different results). We further elaborate on this topic in the discussion.

### Children’s age and face recognition abilities

To explore whether face recognition abilities in children improve with age, a correlation between age and CFMT-K scores for masked and non-masked upright faces was calculated. In line with previous literature, face recognition abilities were positively correlated with age, such that older children performed better on the CFMT-K (masked faces: *r*_(35)_ = 0.57, *p* < 0.001) (Fig. 2c); non-masked faces: *r*_(33)_ = 0.35, *p* = 0.03) (Fig. 2d). Despite the numerical differences, these correlations were not statistically different [*Z* = 1.15, *p* > 0.1].

**Fig. 3:**
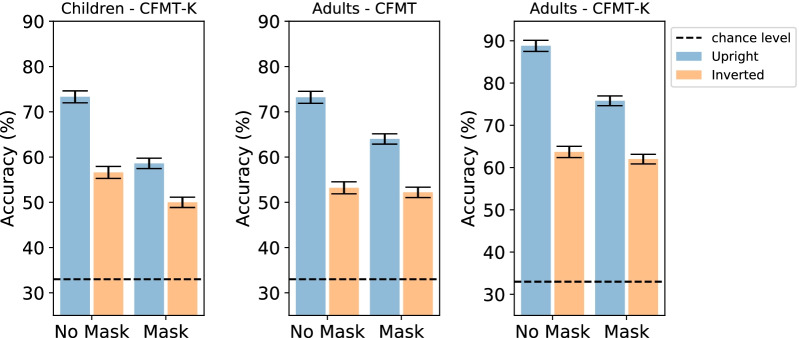
Results of children’s CFMT-K performance, adults’ CFMT performance and adults CFMT-K performance for non-masked and masked faces across orientations. The mask effect found in children was larger than the effect documented in adults who completed the CFMT. Across groups, an inversion effect was found for masked and non-masked faces, but it was significantly reduced for masked faces. Error bars represent the 95% confidence interval for the main effect of group (mask/no mask)

Notably, as mask type (mask/no mask) was manipulated as a between-subjects variable, we could not directly assess the correlation between age and the mask effect. Thus, we split the children into two age groups (11 years and younger and older than 11) and conducted an ANOVA with age group as an additional between-subjects variable. This analysis revealed a robust main effect of age-group with better performance for older children [*F*_(1,68)_ = 21.07, *p* < 0.001, *η*_*p*_^2^ = 0.23] and a two-way interaction between age-group and orientation [*F*_(1,68)_ = 5.27, *p* = 0.025, *η*_*p*_^2^ = 0.072], such that a greater inversion effect was found for older children. This finding might serve as an indication that holistic processing mechanisms are subject to a protracted developmental trajectory.

Importantly, however, we did not find any evidence [*F* < 1] for differences in the effect of mask across the two groups of children [young children—19.7%, older children—22.4% for upright faces]. This result suggests that while face perception abilities are subject to a prolonged developmental trajectory, the mask effect is relatively stable during childhood.

### Children’s and adults’ face recognition performance

Next, we compared children’s face recognition abilities to that of adults. First, we compared children’s performance to that of a group of 495 adults who completed the CFMT with adult upright and inverted masked and non-masked faces. Notably, the two tests are adjusted in terms of their difficulty to account for the differences across the age groups. Hence, the comparison between adults and children can uncover potential differences in the mask effect while controlling other variables.

A repeated measures ANOVA with age group (adult/child), mask type (mask/no mask) and orientation (upright/inverted) was conducted. First, we found that the overall accuracy rate was similar across the two age groups [*F*_(1,563)_ < 1], confirming that the difficulty level was adjusted across the two tests (i.e., CFMT/CFMT-K). Importantly, we found a modest two-way interaction between mask type and age group [*F*_(1,563)_ = 4.82, *p* = 0.028, *η*_*p*_^2^ = 0.008], reflecting a greater mask effect for children (20.1%, upright faces) compared to adults (13.6%, upright faces) (Fig. 3). This finding might suggest that children are more susceptible to the visual alterations embedded in masked faces. Finally, we found an additional two-way interaction between mask type and orientation [*F*_(1,563)_ = 36.44, *p* < 0.001, *η*_*p*_^2^ = 0.06], mirroring the greater inversion effect for non-masked faces. This effect was similar across the age groups, as the three-way interaction was not significant [*F* < 1], suggesting that in both groups holistic processing was disrupted by face masks to a similar extent (Fig. 3). Notably, these results were fully replicated when we used a bootstrap approach to equate the number of participants across the two groups (see Additional file 1: Fig. S1).

An additional challenge to the interpretation of face perception abilities across the two age groups is posed by the use of different versions of the CFMT task (CFMT-K vs. CFMT). Hence, we also tested a group of 72 adults who completed the CFMT-K, thus equalizing the sample size and ensuring that both children and adults are exposed to the same set of face stimuli.

We used a repeated measures ANOVA with gender, age group and mask type, and orientation as independent variables. As expected, we found main effects of gender [females > males; *F*_(1,136)_ = 7.068, *p* < 0.01, *η*_*p*_^2^ = 0.049], mask type [*F*_(1,136)_ = 19.325, *p* < 0.001, *η*_*p*_^2^ = 0.124], and orientation [*F*_(1,136)_ = 198.7, *p* < 0.001, *η*_*p*_^2^ = 0.594]. Since difficulty was no longer adjusted across age group, we also found a robust main effect of age group [*F*_(1,136)_ = 33.98, *p* < 0.001, *η*_*p*_^2^ = 0.2], demonstrating a clear advantage in face perception abilities for the adult group (Fig. 3).

In addition to these main effects, we also found a two-way interaction between orientation and mask type (i.e., reduced inversion for the mask condition; [*F*_(1,136)_ = 17.99, *p* < 0.001, *η*_*p*_^2^ = 0.117]). The masked faces condition elicited a smaller inversion effect in the adult group, but this reduction could not be attributed to a floor effect, as adults performed reasonably well even for masked inverted faces (~ 60%). We also found a two-way interaction between orientation and age group, such that adults exhibited a greater inversion effect [*F*_(1,136)_ = 9.066, *p* < 0.01, *η*_*p*_^2^ = 0.062] pointing to a greater degree of holistic processing for adults. The three-way interaction between group, orientation, and mask type was not significant [*F*_(1,136)_ < 1], suggesting that the reduced inversion effect for masked faces was similar across age groups.

Finally, we did not find evidence for differences in the size of the mask effect between the two groups [*F*_(1,136)_ < 1]. The absence of this effect might be accounted for by the robust differences in the overall performance levels observed for the two age groups (i.e., adults = 72.5%; children = 59.5%). Another related explanation for the lack of interaction is a celling effect for the upright, non-masked faces for the adult group (accuracy ~ 90%, with 17 out of 36 participants with a performance level greater than 95%), further emphasizing the importance of adjusting performance difficulty between children and adults.

## Discussion

Face masks have been accepted as an important tool to minimize the spread of COVID-19 and are thus prevalent in everyday social interactions. In the current study, we evaluated whether school-age children demonstrate a similar impairment in face perception abilities caused by face masks as previously found in adults (Carragher & Hancock, 2020; Freud et al., 2020). We have documented quantitative and qualitative changes in face processing abilities for masked faces in children. In particular, face masks led to a robust decrease in face processing abilities measured by the CFMT-K. This quantitative reduction was accompanied by a reduced inversion effect for masked faces, suggesting a qualitative change in the way masked faces are processed. The reduction of the FIE for masked faces was similar in younger and older children, implying that holistic face processing is similarly disrupted across ages.

The size of the mask effect was compared between children and two separate groups of adults. First, we compared the children to adults who completed the CFMT-K and the CFMT, thus equalizing the overall level of performance across the groups. Under this condition, children showed a greater mask effect (20.1% compared to 13.5% for adults), suggesting greater susceptibility to visual alterations caused by face masks. These findings were maintained when sample size between the adults and children groups was adjusted via a bootstrap analysis. Next, we compared the children to adults who completed the CFMT-K and found a similar mask effect for both groups. Notably, however, the adults outperformed children in their overall performance, and this robust difference (together with a plausible celling effect) might hinder our ability to identify any changes in the size of the mask effect. Taken together, we propose that (a) it is plausible that the effect of masks on face perception abilities might be slightly greater for children and (b) any comparison between perceptual abilities of children and adults needs to take into account the overall level of performance across age groups.

### Reduced holistic processing for masked faces

The current experiment also provides evidence for a reduction of the face inversion effect for masked faces in children. Specifically, for non-masked faces we found a decrease of 23% in the CFMT-K score for inverted faces, while a smaller inversion effect of 15% was found for masked faces. Notably, this effect could not be attributed to a floor effect, because children were well above chance level even for the masked, inverted, condition. The inversion of a face makes it difficult to extract configural relationships between face features (Farah et al., 1995; Freire et al., 2000; Yin, 1969); therefore, the twofold smaller inversion effect for masked faces can be taken as evidence that holistic face processing is largely reduced, though not entirely abolished. Thus, the processing of masked faces relies more heavily on the available features rather than on configural or holistic information.

The inversion effect is typically suggested to reflect a reduction in holistic processing and greater reliance on sequential, spatially restricted processing of face features (Rossion, 2009). This view can account for the smaller inversion effect for masked faces. In particular, the upright masked faces are processed in a less holistic manner, resulting in reduced face perception abilities. Then, when the masked faces are inverted, the effect of the mask is less evident due to feature processing being spatially limited, thus leading to a reduced face inversion effect. A similar alteration of face perception and holistic processing has been documented within the context of the “other race effect” (ORE; Kuefner et al., 2010; Mondloch et al., 2007). Reduced face recognition performance in these studies was interpreted as evidence for reduced holistic processing of other-race faces. Together, these findings provide evidence for the co-occurrence of a reduction in face perception abilities and a disruption of holistic face processing.

Interestingly, the face inversion effect increased for older children, presumably reflecting a greater reliance on holistic processing in this group. Along similar lines, a greater inversion effect was found among the adults who completed the CFMT-K. Hence, if the mask effect solely reflects a disruption in holistic processing, a plausible prediction would be that younger children should exhibit a reduced mask effect. However, this was not the case, as the mask effect remained stable across children’s ages. This pattern of results suggests that the mask effect is more likely to reflect a reduction in both holistic and featural processing. The relative contribution of each of those components might change throughout development and requires further research.

### Sex/gender differences in face perception abilities

An additional finding of the current study was better face recognition performance for female compared to male children. Superior face perception abilities in females has been extensively documented in adult participants (Bai et al., 2015; Bobak et al., 2016; Freud et al., 2020; McBain et al., 2009); however, findings in the developmental literature are less consistent. One study has reported a strong overall face recognition advantage for female children, with a magnified effect for own-sex faces (Rehnman & Herlitz, 2006); however, others have found only a minimal effect of sex/gender on face perception, with girls performing better on old/new and face inversion tasks (Zhu et al., 2010).

One limitation of the present study is the exclusive use of male faces in the CFMT-K. It is possible that greater sex/gender diversity in the face stimuli set would result in an even greater sex/gender difference between males and females than currently observed, given documented face recognition advantages for own-sex faces (Rehnman & Herlitz, 2006). On a similar note, it worth mentioning that the CFMT / CFMT-k suffers from the lack of ethnic diversity as only Caucasian faces were included. Hence, future studies should use the CFMT-K with a combination of male and female faces and ethnically diverse faces to explore possible sex/gender differences and the ORE in face recognition.

## Conclusion

The current study provides evidence for quantitative and qualitative changes in the processing of masked faces in children. Changes in face recognition performance and alteration in the processing of partially occluded faces could have significant effects on children’s social interactions with their peers and their ability to form relationships with educators. Previous research in adults has already demonstrated the detrimental effect of reduced face perception abilities on one’s level of social confidence and quality of life (Lane et al., 2018). Given the recent increased uptake in mask-wearing due to the COVID-19 pandemic, future research should explore the social and psychological ramifications of wearing masks on children’s performance.

## Supplementary Information

**Additional file 1.** Bootstrap analysis demonstrated that children show a larger mask effect even when sample size is taken into consideration.

## Data Availability

Data and analysis code are available on the Open-Source Framework (https://osf.io/yj38h/) under CC-By Attribution 4.0 International license.
