# Supplementary material for: Face masks disrupt holistic processing and face perception in school-age children
Source: Cogn Res Princ Implic. 2022 Feb 7;7:9. doi: 10.1186/s41235-022-00360-2 (PMC8818366; doi:10.1186/s41235-022-00360-2)
Supplement: Supplementary file 1 — Additional file 1. Bootstrap analysis demonstrated that children show a larger mask effect even when sample size is taken into consideration. [file 41235_2022_360_MOESM1_ESM.docx]

**Supplementary Material:**

**Face Masks Disrupt Holistic Processing and Face Perception in School-Age Children (Stajduhar et al)**

One limitation of the comparison between the children and adults groups is the large difference in sample sizes (495 adults compared to 72 children). To address this discrepancy, we conducted a bootstrap analysis. In each iteration (n=1000), we randomly sampled (without replacement) 72 adult participants and calculated the mask effect (across the inverted and upright conditions). This approach yielded a bootstrap distribution for which we computed the 95% confidence intervals. We then compared the mask effect observed for children to the observed bootstrap distribution.

As demonstrated in Figure 1S, the mask effect observed for children was greater than the average mask effect of the bootstrap distribution generated from the adult sample [bootstrap average: 8.4% CI: 2.83%-13.9% Children percentile: 16.5%]. Finally, we repeated this process only for upright faces and found similar results (i.e., greater mask effect for children; bootstrap average: 13.5% CI: 6.9%-19.9% Children percentile: 20.05%; Fig. 1S). The results from the bootstrap analysis suggest that children show a larger mask effect even when sample size is taken into consideration (Fig. 4b).

**Fig.** **1S** Bootstrap distribution of mask effect across the (a) inverted and upright conditions and (b) the upright condition, generated from a random sample of 72 adults (1000 iterations). The red dot represents children’s mask effect for the same conditions. The dashed vertical lines show the confidence interval (2.5%, 97.5%) of the bootstrap distribution. A greater mask effect was observed for children, even when sample size was adjusted
